# The Effects of Light upon Chloroform—Important Suggestion to Those Using the Article

**Published:** 1868-02

**Authors:** Wm. Jay Youmans


### THE EFFECTS OF LIGHT UPON CHLOROFORM—IMPORTANT SUGGESTION TO THOSE USING THE ARTICLE.

BY WM. JAY YOUMANS, M. D.

During the progress of the Coroner's investigation in the recent case of death from chloroform in this city, (Winona, Wis.,) several questions of more than transient importance came up for consideration. Among these none possessed greater interest than the two following: First,—Will light decompose chloroform? Second,—If so, what length of time is required to render the decomposition apparent? In regard to the first question, the uniform reply of the books was that it will. Touching the second the books were entirely silent; and in the absence of any personal knowledge upon the part of the several gentlemen called as witnesses, the question was left wholly unanswered. In view of this unsatisfactory state of the matter, and in response to the wishes of a number of gentlemen who were connected with the case, I am led to give publicity to the following occurrence.

It will be remembered that at the time the chemical examination of the chloroform was going on, a fresh bottle, represented as belonging to the same invoice as that used by Dr. Welch, was obtained from Mr. Wickersham, and also subjected to examination. This presented nothing extraordinary in appearance, and was found to be perfectly pure. At the close of the inquest it was left standing upon the table, near a window, in a private room in my office, where, for a portion of the day—from about nine o'clock until twelve—it was exposed to the direct rays of the sun. I took no particular notice of it further than an occasional glance, until one day—the twenty-fifth, I think, from the time of bringing it to the office—I observed upon the bottom of the inside of the bottle a greenish appearance which at once arrested my attention. Struck by the similarity of color between it and the foreign substance present in the chloroform which came from the office of Dr. Welch, I immediately dipped into the bottle a slip of test-paper, and found the contents to be distinctly acid. A day or two after I observed that a greenish colored fluid was collecting upon the surface of the chloroform; and this has since gone on accumulating until now it forms a kind of ring nearly a quarter of an inch thick, adhering to the sides of the bottle, and floating upon the surface. I have tested this chloroform carefully, and find the new substance to be hydrochloric acid.

As stated before, the several works which I have been able to consult upon the point, all of which are authoritative, say that light will decompose chloroform, and that hydrochloric acid is one of the most common products of this decomposition. That this change may take place rapidly under favorable conditions of exposure, and thus render the chloroform highly dangerous, and totally unfit for use, is a conclusion that seems to be fully warranted from the foregoing facts.

